# Fulminant serogroup B meningococcemia in an adolescent vaccinated against serogroups A and C: highlighting the serogroup B vaccination gap in China—a case report and literature review

**DOI:** 10.3389/fmed.2026.1818444

**Published:** 2026-10-07

**Authors:** Qifa Hu, Jun Jiang, Haixia Zhu, Zhenzhu Yao, Shanghong Tang, Hongxing Li, Weipeng Feng, Xuefei Zeng, Xiuling Huang, Hailing Huang, Dan Zhang

**Affiliations:** 1 Department of Pediatrics, Huidong County People's Hospital, Huizhou, Guangdong, China; 2 Pediatric Intensive Care Unit, Huizhou Central People's Hospital, Huizhou, Guangdong, China; 3 Department of Laboratory Medicine, Huidong County People's Hospital, Huizhou, Guangdong, China; 4 Department of Gastroenterology, Henan Children's Hospital Zhengzhou Children's Hospital, Henan, China

**Keywords:** adolescent, meningococcemia, Neisseria meningitidis, purpura fulminans, serogroup b

## Abstract

**Background:**

Invasive meningococcal disease (IMD) remains an acute clinical challenge associated with high mortality and permanent disability. Although routine immunization has successfully suppressed historical lineages, an expanding epidemiological shift toward serogroup B in specific regions has exposed a population vulnerability, given that protein-based serogroup B vaccines are excluded from China's National Immunization Program (NIP, historically referred to as EPI). We present a case that characterizes the clinical trajectory of this vaccination mismatch.

**Case presentation:**

We report a case of a 12-year-old boy from Guangdong Province, China, who had previously received vaccination against Neisseria meningitidis serogroups A and C. He presented with fever and a rapidly progressive purpuric rash. Within hours, his condition deteriorated to septic shock, disseminated intravascular coagulation (DIC), and multiple organ dysfunction syndrome (MODS), consistent with purpura fulminans. Blood cultures confirmed N. meningitidis serogroup B. Following aggressive intensive care support—including antibiotics, vasopressors, mechanical ventilation, plasma exchange, and continuous renal replacement therapy—the patient survived, exhibiting complex ischemic sequelae that secondary cutaneous necrosis necessitated multi-stage surgical autologous grafting, which subsequently resulted in long-term functional extremity deficits.

**Conclusion:**

This case underscores the hyperacute progression of serogroup B meningococcemia in an adolescent previously immunized against serogroups A and C. The clinical course illustrates an individual vulnerability under the current polysaccharide-based framework and highlights the public health relevance of reassessing regional vaccine strategies and enhancing surveillance for emerging serogroups.

## 1 Introduction

*Neisseria meningitidis* remains a leading cause of bacterial meningitis and sepsis worldwide. Invasive meningococcal disease (IMD), encompassing meningitis, meningococcemia, or both, is notorious for its abrupt onset, rapid clinical deterioration, and potential for devastating outcomes. Despite advances in vaccination and critical care, IMD continues to carry a mortality rate of 10%–20%, with up to 20% of survivors experiencing permanent sequelae such as hearing loss, neurological deficits, or limb amputations (1, 2, 33).

In China, concerted public health efforts have dramatically reduced the national incidence of IMD. However, the epidemiological landscape is shifting. While serogroup A was historically dominant, serogroup C has emerged as the predominant cause of IMD in recent years (3, 4). More concerning, sporadic cases caused by serogroups B and W have been increasingly documented, particularly in Guangdong Province (3, 5). This epidemiological transition creates a vulnerable population when local immunization programs fail to cover these emerging serogroups.

A significant barrier to comprehensive prevention lies in the unique nature of serogroup B. Its capsular polysaccharide, composed of polysialic acid, is structurally identical to human neural cell adhesion molecules. This molecular mimicry results in poor immunogenicity and a risk of autoimmunity, which historically hindered the development of traditional polysaccharide-conjugate vaccines effective against other serogroups (6). Consequently, despite the global availability of protein-based serogroup B vaccines, they remain outside China's National Immunization Program, creating a critical vulnerability.

The most critical presentation of IMD is fulminant meningococcemia complicated by purpura fulminans (PF), a clinical syndrome characterized by disseminated microvascular thrombosis, diffuse cutaneous hemorrhage, and rapid circulatory collapse (7, 34). Early diagnosis is frequently confounded by the non-specific nature of prodromal constitutional symptoms, which mirror benign viral syndromes. This clinical overlap introduces a significant risk of diagnostic delay, particularly in low-incidence settings where frontline clinicians maintain a low index of suspicion.

To contextualize the management challenges and long-term morbidity associated with severe meningococcemic purpura fulminans, a targeted narrative literature search was executed across PubMed, Embase, Web of Science, and Chinese national databases (CNKI and SinoMed) for studies published between January 1, 2014, and December 31, 2025. The verbatim Boolean search string utilized was: [“Neisseria meningitidis”(Mesh) OR “meningococcemia”] AND (“purpura fulminans” OR “septic shock”) AND (“serogroup B” OR “MenB”). Search parameters were restricted to English and Chinese peer-reviewed publications.

This structured inquiry initially yielded 134 records across the combined databases. We implemented a two-stage screening process: an initial title/abstract evaluation to exclude broad epidemiological registries lacking individual intervention data, followed by a rigorous full-text appraisal of 28 remaining candidates against specific inclusion criteria (laboratory-confirmed N. meningitidis, severe PF/septic shock requiring advanced organ support, and detailed long-term outcome tracking). Ultimately, 7 representative landmark studies and case series fulfilling all parameters were selected for structural comparison in Table 2. The complete study selection process is illustrated in the PRISMA-style flow diagram (Figure 1).

**Figure 1:**
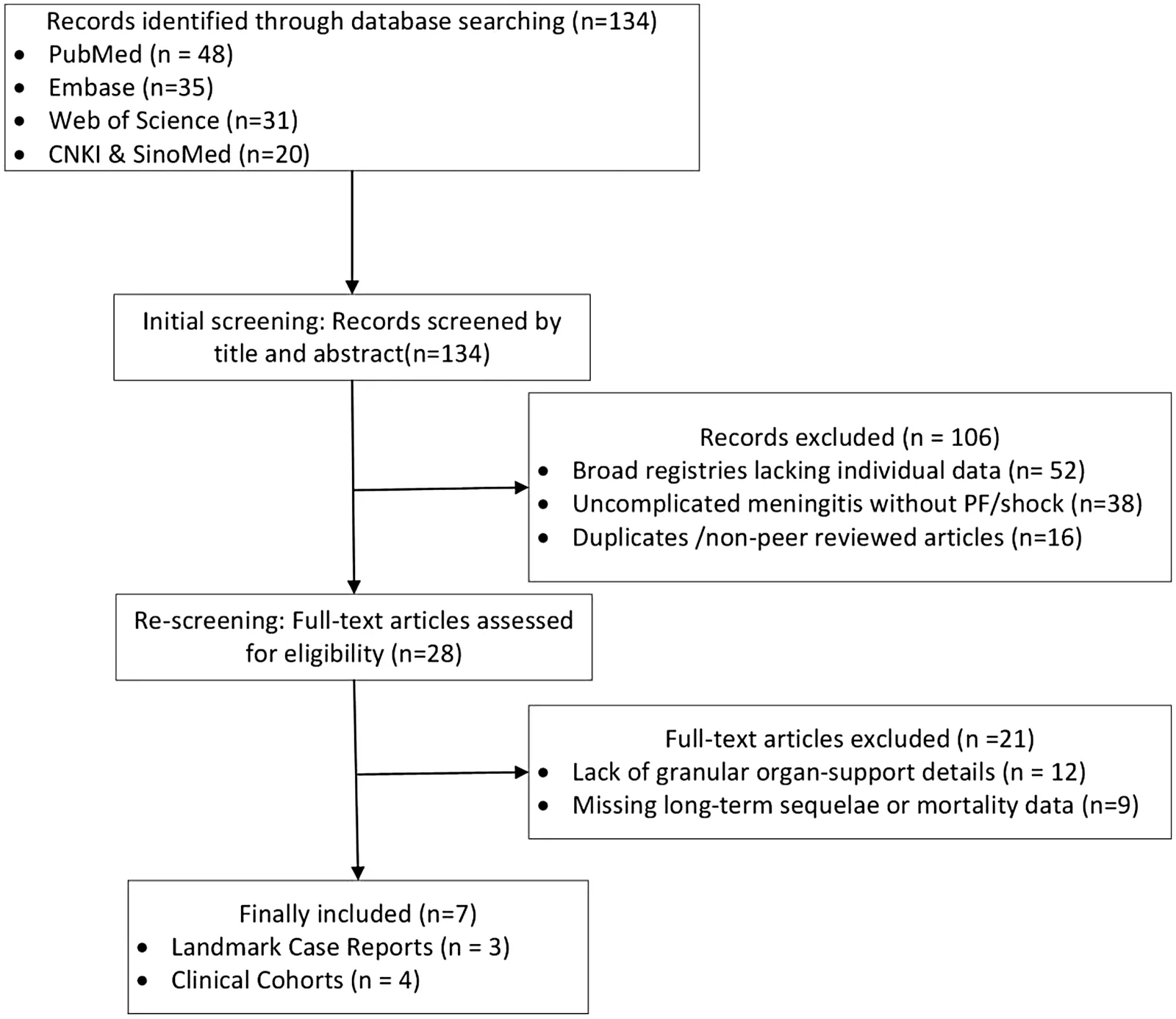
PRISMA-style selection flow diagram for targeted literature review. Illustrates the initial search output (*n* = 134), primary screening exclusion (*n* = 106), full-text appraisal (*n* = 28), and final selection of 7 representative landmark cohorts and case series for structural synthesis in Table 2.

## 2 Case description

A previously healthy, fully immunized 12-year-old boy (with documented receipt of serogroup A/C polysaccharide vaccines) presented with a rapidly deteriorating febrile illness. To clarify the clinical timeline, time points are anchored to the initial Emergency Department (ED) presentation at the primary hospital (Hour 0):

Hour -18 (Pre-admission): Onset of fever (peak 38.3 °C) and mild upper respiratory symptoms. Managed at an outpatient clinic with oral cefradine and antipyretics under a presumptive diagnosis of acute viral infection.

Hour -10 (10 h pre-ED): First appearance of scattered purpuric lesions on the neck and lower extremities. This warning sign was documented but did not prompt immediate emergency referral.

Hour -7 (7 h pre-PICU): Presentation to the county emergency department. The patient was hemodynamically stable and alert. Laboratory evaluation revealed a mild CRP elevation (30.64 mg/L) with normal platelet count and coagulation profile (Figure 2; Table 1). The cutaneous manifestations were misattributed to acute gastritis with allergic/benign purpura, and supportive IV fluids were initiated.

**Figure 2:**
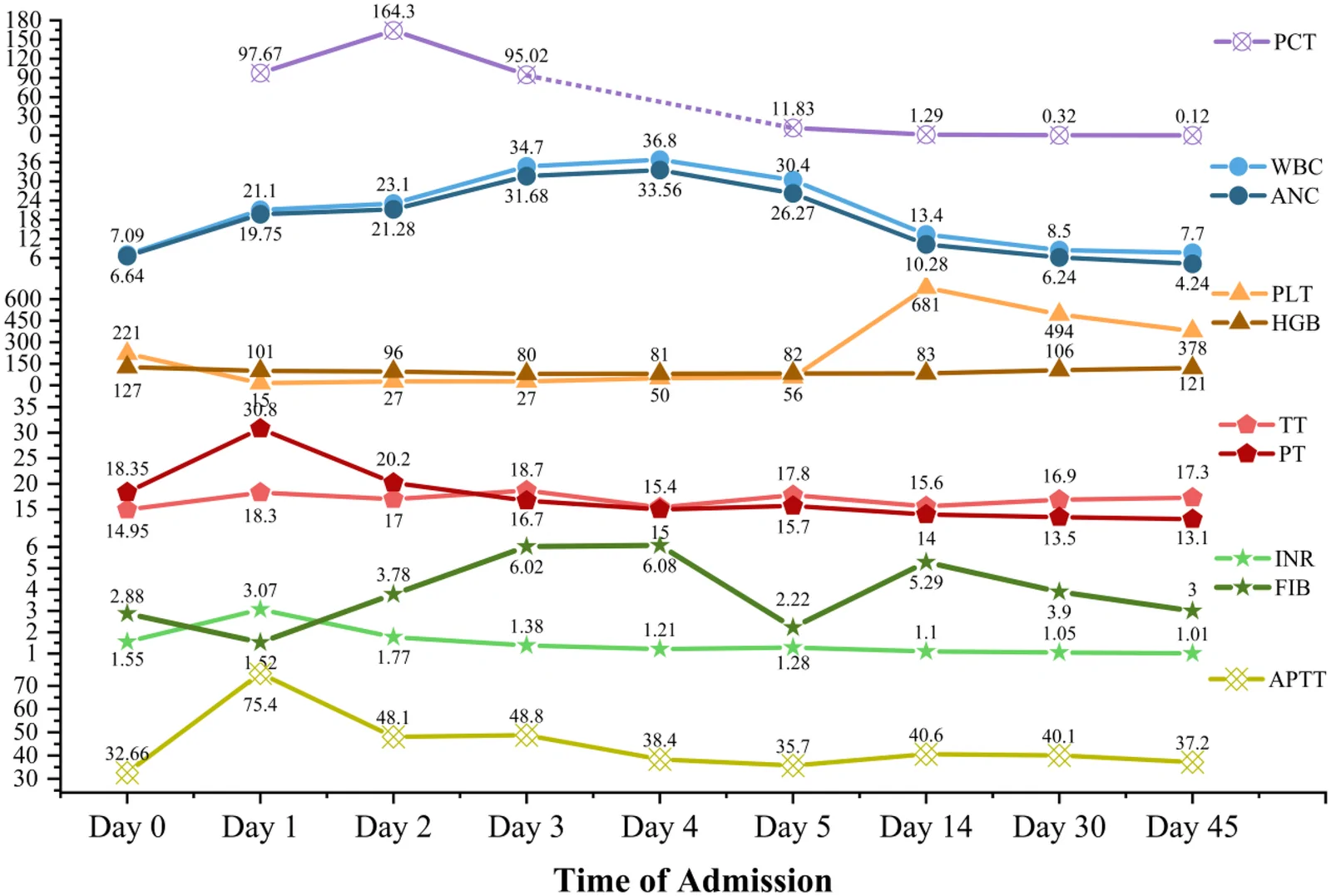
Temporal trends of key laboratory parameters. Day 0, Pre-admission. PCT, Procalcitonin. WBC, White blood cell. ANC, Absolute neutrophil count. PLT, Platelet. HGB, Hemoglobin. TT, Thrombin time. PT, Prothrombin time. INR, International normalized ratio. FIB, Fibrinogen. APTT, Activated partial thromboplastin time.

**Table 1:** Reference ranges for laboratory parameters.

Laboratory Parameter	Unit	Reference Range
PCT	ng/mL	0−0.046
WBC	10^9^/L	4.3−11.3
ANC	10^9^/L	1.6−7.8
PLT	10^9^/L	150–407
HGB	g/L	118–156
TT	s	14–21
PT	s	11–14.5
INR	/	0.8–1.2
FIB	g/L	2.0–4.0
APTT	s	28–41

WBC, white blood cell. ANC, absolute neutrophil count. PLT, platelet. HGB, hemoglobin. PCT, procalcitonin. TT, thrombin time. PT, prothrombin time. INR, international normalized ratio. APTT, activated partial thromboplastin time. FIB, fibrinogen. s, second.

Hour -7 to Hour 0: Over the next 7 h, the purpura rapidly coalesced into painful, confluent ecchymoses with central hemorrhagic necrosis, accompanied by persistent fever and severe leg pain (Figure 3).

**Figure 3:**
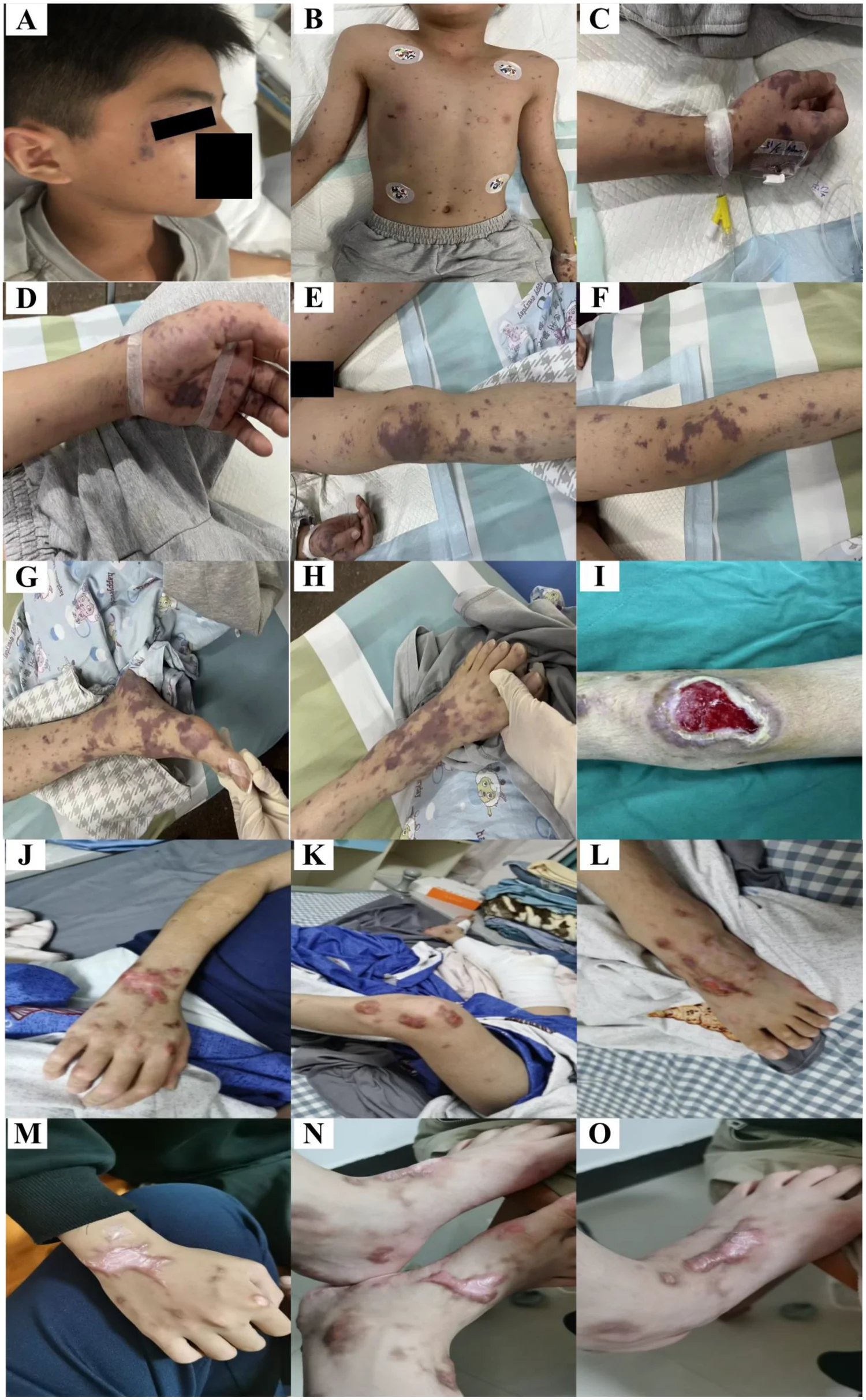
Clinical course of skin lesions. **(A–I) Acute phase. (A–H)** Progressive purpuric rash and ecchymoses covering the body. (I) Intraoperative view of necrotic skin on the right knee during debridement and grafting. **(J–O) FolloW,Up. (J–L)** Hypertrophic scars on the limbs at four months. **(M–O)** Persistent scarring and limited mobility of the grafted right knee at six months.

Hour 0 (PICU Admission): Admitted to the pediatric ward and rapidly transferred to the PICU upon progression to refractory septic shock (BP 80/50 mmHg, HR 130 bpm, CRT 3 s), severe lethargy, and disseminated intravascular coagulation (DIC; platelets 68  ×  10⁹/L, INR 1.79, D-dimer 33.99 mg/L). The total duration from initial purpura appearance to PICU critical care admission spanned 17 h.

Upon PICU arrival, the patient's condition acutely deteriorated to multiple organ dysfunction syndrome (MODS), with refractory shock (lactate 10.5 mmol/L), severe thrombocytopenia (15 × 10⁹/L) (Figure 2), and profound cardiac depression (LVEF 20%). Aggressive multimodal support was immediately instituted: mechanical ventilation, high-dose vasopressors, broad-spectrum antibiotics (meropenem plus vancomycin), and early initiation of therapeutic plasma exchange (TPE) and continuous renal replacement therapy (CRRT) to modulate the inflammatory cascade and support organ function (Figure 4).

**Figure 4:**
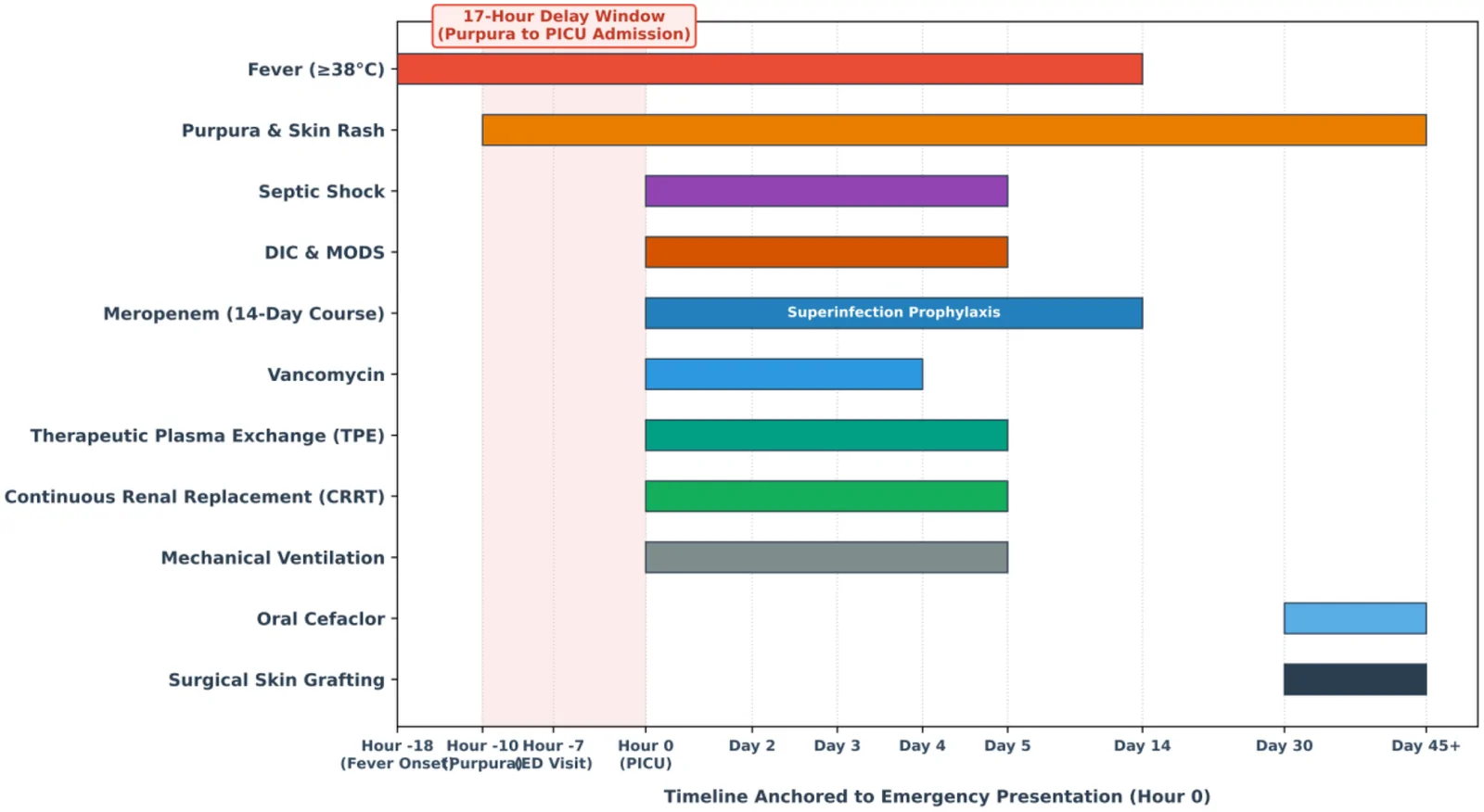
Schematic timeline of clinical manifestations and therapeutic interventions. MODS, multiple organ dysfunction syndrome; DIC, disseminated intravascular coagulation; CRRT, Continuous Renal Replacement Therapy. The extended 14-day course of meropenem was indicated as empiric prophylaxis against secondary bacterial superinfection across denuded necrotic skin prior to grafting, rather than delayed N. meningitidis clearance.

Blood cultures confirmed N. meningitidis serogroup B on day 4 (Figure 5). The patient's remarkable recovery of cardiac function (EF 52% by day 3) and shock reversal by day 5 demonstrate the potential reversibility of even severe septic cardiomyopathy when aggressive supportive care is deployed early.

**Figure 5:**
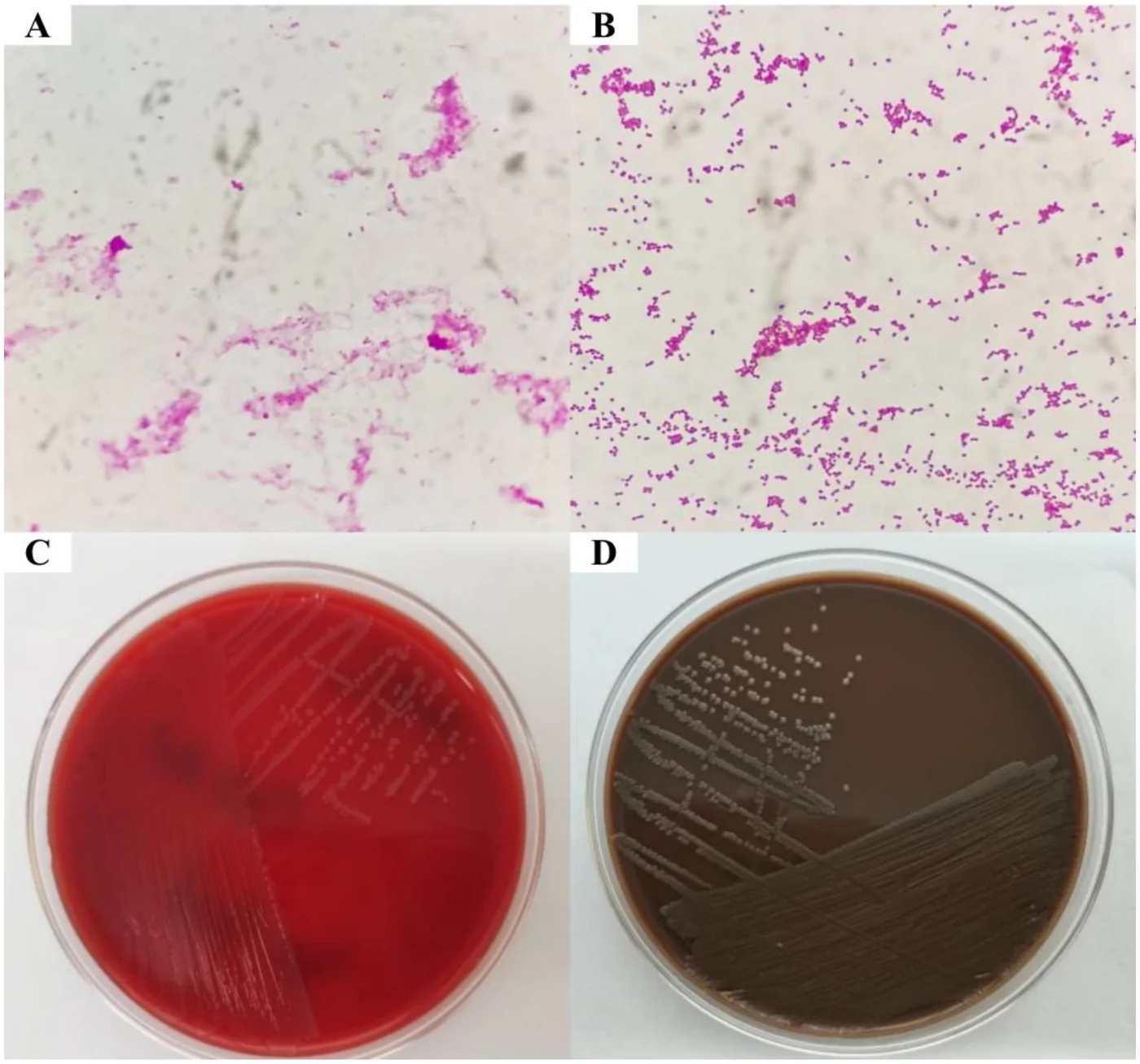
Microbiological identification of Neisseria meningitidis serogroup B. **(A)** Direct Gram stain of the positive aerobic blood culture broth, demonstrating characteristic kidney-shaped, Gram-negative diplococci arranged in pairs. **(B)** Gram stain of subcultured colony smear confirming typical diplococcal morphology. **(C,D)** Colony morphology after 24 h of incubation at 35 °C in a 5% CO_2_ atmosphere: **(C)** raised, moist, grayish translucent colonies on blood agar; **(D)** moist, dewdrop-like colonies on chocolate agar. Serogroup B confirmation was definitively established on Hospital Day 4.

The patient survived with significant morbidity: extensive skin necrosis required surgical debridement and skin grafting, resulting in permanent hypertrophic scarring and residual right knee contracture with a persistent limp at follow-up.

## 3 Discussion

This case starkly illustrates the significant clinical challenge of fulminant meningococcemia, demonstrating its capacity to progress from benign symptoms to multi-organ failure within hours. The discussion that follows integrates the lessons from this case with a review of the relevant literature, focusing on clinical challenges, epidemiological shifts, therapeutic imperatives, and long-term outcomes.

### 3.1 Clinical presentation, diagnostic pitfalls, and pathophysiology of purpura fulminans

The initial presentation of invasive meningococcal disease (IMD) closely mimics benign viral illnesses, which significantly increases the risk of diagnostic delay. Our patient's trajectory—from an initial diagnosis of “acute upper respiratory infection” to fulminant septic shock within 18 h—exemplifies this hyperacute or atypical progression. Recent evidence underscores that clinical prediction rules based solely on early symptomatology exhibit limited sensitivity, and the absence of classic meningeal signs does not exclude severe disease (15). The rapid evolution from petechiae to confluent purpura and ecchymosis should serve as an unequivocal red flag; however, as illustrated in this case, initial laboratory findings—including platelet count and conventional coagulation parameters—may remain within normal limits, offering a misleading assessment of disease severity. Emerging biomarkers, such as procalcitonin and host gene-expression signatures, hold promise for earlier discrimination, but they are not yet routinely decisive in acute care settings (16).

Purpura fulminans (PF) represents the most severe phenotypic extreme of this disease spectrum. Its pathophysiology is driven by a catastrophic interplay among infection, systemic inflammation, and coagulation dysregulation. Bacterial endotoxin release triggers an overwhelming systemic inflammatory response, culminating in a “cytokine storm” (17). This cascade inflicts endothelial injury, activating coagulation while suppressing endogenous anticoagulant pathways—most notably protein C and thrombomodulin (12). The resultant DIC promotes microvascular thrombosis, tissue ischemia, and hemorrhagic skin necrosis.

Crucially, clinical evidence demonstrates that hyperacute mortality in meningococcal PF occurs predominantly within the first 24–72 h due to refractory endotoxic shock and myocardial failure; patients who survive past this initial 72-hour window rarely succumb to shock itself, but instead face severe complications of microvascular thrombosis, tissue necrosis, and secondary superinfections (18). The 17-hour delay from initial purpura emergence to PICU transfer in our patient highlights the vital importance of early clinical recognition during the narrow therapeutic window.

### 3.2 the shifting epidemiology of N. meningitidis in China

The global landscape of meningococcal disease is in constant flux due to natural variation, vaccine pressure, and human mobility. In China, robust surveillance data confirms a decisive epidemiological shift over the past decade. While vaccination efforts successfully controlled serogroup A, serogroup C emerged as the dominant cause of IMD (3). More recently, serogroups B and W have risen in prominence, accounting for a significant and growing proportion of cases in several provinces (5, 19).

According to national surveillance data, serogroup B accounted for 67.29% of typed meningococcal meningitis cases among Chinese infants during 2019–2023 (up from 14.55% in 2006–2010) (35). Concurrently, provincial surveillance in Zhejiang (2014–2024) demonstrated that serogroup B constituted 53.85% (21/39) of all-ages typed isolates (36). In Guangdong Province, where our patient resided, Wang et al. (5) reported that among 278 IMD cases, serogroup C accounted for 41%, serogroup B for 32%, and serogroup W for 15%. Rather than representing a single uniform rate, these distinct surveillance strata collectively demonstrate a high and rising prevalence of serogroup B across multiple epidemiological cohorts in mainland China.

### 3.3 Serogroup B coverage, molecular considerations, and vaccine immunogenicity

China's National Immunization Program (NIP) currently includes only serogroup A polysaccharide vaccine (administered at 6 and 9 months) and serogroup A + C polysaccharide vaccine (administered at 3 and 6 years), leaving a complete absence of coverage for serogroup B (20, 21, 42). Protein-based vaccines such as 4CMenB (Bexsero) have demonstrated 71%–95% real–world effectiveness in international programs (22–24, 38, 39), yet remain unlicensed in mainland China.

In evaluating this patient's susceptibility, the potential waning of polysaccharide-induced immunity must be considered alongside the lack of serogroup B vaccine coverage. The patient received his final dose of A + C polysaccharide vaccine at 6 years of age—approximately 6 years prior to the current presentation. Because plain polysaccharide vaccines do not induce robust immunological memory or long-term mucosal immunity, bactericidal antibody titers naturally decline over time. Thus, while the lack of serogroup B coverage was the primary driver of disease from an uncovered strain, waning A/C immunity represents a co-existing factor in overall host vulnerability.

Furthermore, the prospective clinical utility and cross-protection of protein-based vaccines like 4CMenB depend on target antigen expression (fHbp, NadA, NHBA, and PorA). In China, molecular surveillance indicates that the dominant circulating serogroup B lineage belongs to the clonal complex 4821 (CC4821), which exhibits antigenic divergence from Western reference strains (4, 21). Because isolate-level whole-genome sequencing (WGS) and Meningococcal Antigen Typing System (MATS) assays were not performed on the recovered strain in this patient, this case cannot directly prove antigenic mismatch. Rather, it serves as a clinical sentinel that raises the question of vaccine-matching and highlights the need for systematic MATS indexing of domestic isolates.

### 3.4 Evolving strategies in critical care management

The management of fulminant meningococcemia demands immediate, multimodal intervention in a pediatric intensive care unit (PICU). The cornerstone remains early, high-dose bactericidal antibiotic therapy. Third-generation cephalosporins (e.g., ceftriaxone) are the empiric gold standard, though carbapenems (e.g., meropenem) are used in settings of high resistance prevalence or severe penicillin allergy (10). The critical importance of minimizing the time to first antibiotic dose cannot be overstated, as delays are linearly associated with increased mortality (25).

Beyond prompt antimicrobial initiation, aggressive extracorporeal support represents a cornerstone in mitigating the systemic sequels of meningococcal-induced PF (8). The hyperacute phase of this condition is governed by an overwhelming endotoxin-driven cytokine storm that triggers profound endothelial cytotoxicity and a devastating consumption coagulopathy, characterized by the catastrophic depletion of endogenous anticoagulant substrates including protein C, protein S, and antithrombin III (12, 17). In this critical dynamic, therapeutic plasma exchange (TPE) exerts a dual pathophysiological benefit: it facilitates the literal macro-clearance of circulating lipooligosaccharide (LOS) endotoxins and pro-inflammatory immune complexes, while simultaneously delivering therapeutic volumes of fresh frozen plasma to actively replenish depleted natural coagulation inhibitors and halt microvascular thrombosis (11, 26, 40). Concurrently, the integration of continuous renal replacement therapy (CRRT) provides essential metabolic homeostasis and metabolic clearance during acute septic cardiomyopathy and subsequent renal hypoperfusion (27).

Regarding antimicrobial stewardship, standard uncomplicated N. meningitidis bacteremia typically mandates a 5-to-7-day course of directed beta-lactam therapy (10). However, the presence of widespread ischemic cutaneous necrosis introduces distinct clinical management paradigms (18, 43). In the present case, the administration of intravenous meropenem was maintained for a total of 14 days. This extended duration was not targeted at the secondary clearance of the primary meningococcal organism—which was sterilized rapidly following critical care admission—but was strictly indicated as an empiric prophylaxis against secondary bacterial superinfection of the extensive, denuded necrotic skin landscapes prior to definitive surgical debridement and autologous skin grafting. The subsequent step-down to oral cefaclor served as intermediate antimicrobial coverage during the hyperacute phase of early surgical wound healing.

### 3.5 Long-term sequelae, survivorship, and integrated public health strategy

Survival is merely the first hurdle. The long-term burden of severe IMD is profound and multidimensional. As seen in our patient, extensive skin necrosis often requires multiple surgical procedures, including debridement and grafting, and can result in permanent scarring, contractures, and limb dysfunction (28). Neurological sequelae, including hearing loss (sensorineural in up to 10% of survivors), cognitive deficits, and motor impairments, are common and can significantly impact quality of life and educational attainment (29, 41). Psychological sequelae, such as post-traumatic stress disorder (PTSD), anxiety, and depression, are increasingly recognized in both survivors and their families (30).

This case compels a dual-focused public health response. First, at the clinical frontier, education must emphasize that a petechial rash in a febrile patient is a medical emergency until proven otherwise. Second, and crucially, immunization policy must be dynamic and evidence-based. Static vaccination programs that do not adapt to changing serogroup epidemiology will inevitably develop gaps. The integration of multivalent conjugate vaccines (MenACWY) and the consideration of introducing protein-based MenB vaccines for high-risk groups or regions with documented serogroup B circulation are essential steps forward (9, 31). Enhanced, real-time surveillance of circulating strains is the bedrock upon which such informed policy decisions must be built. The goal must shift from merely preventing death to preventing the disease altogether and ensuring that survivors have access to the multidisciplinary rehabilitation needed to maximize their long-term well-being.

### 3.6 Synthesis of comparative literature findings

Cross-referencing our patient's clinical trajectory with published cohorts in Table 2 reveals key insights regarding survival and morbidity. While pediatric case reports (Özdemir et al., Inceli et al.) document rapid mortality despite intensive care, our patient survived multi-organ failure, aligning with survival cohorts reported by Bendapudi et al. (11, 13, 28). Notably, while large adult cohorts (Contou et al.) report a 41.2% mortality rate and high rates of major limb amputations, our patient achieved survival with 100% limb preservation, converting severe microvascular ischemia into superficial necrosis manageable by grafting (8, 32).

**Table 2:** Summary of reported cases and series of severe meningococcemia and purpura fulminans.

Author & Year	Country	Study type	Number of cases (Age Range)	Predominant Serogroup(s)	Key clinical features	Major interventions	Outcomes & Sequelae	Case severity
Özdemir et al., 2012 (8)	Turkey	Case report	1 (6-month female)	Not specified	PF, shock, DIC, multiorgan failure	Antibiotics, vasopressors, ventilation	Fatal.	Severe only
Viner et al., 2012 (9)	UK	Case-control (MOSAIC study)	245 children (0–18 yrs)	B (71%), C, W, Y	Meningitis, septicemia, PF.	Standard hospital care.	Case-fatality 6.5%. 13% had major sequelae (hearing loss, neurodisability, amputation).	Mixed
Bendapudi et al., 2018 (10)	USA	Case report	1 (14-year male)	Not specified	Severe PF with protein C depletion	Antibiotics, protein C concentrate, PLEX	Survival with extensive skin necrosis requiring grafting.	Severe only
Contou et al., 2018 (11)	France	Retrospective cohort	306 adults	Not specified (Nm leading cause)	PF, septic shock, DIC, MODS	Antibiotics, organ support, some received PLEX	Mortality 41.2%. Survivors often had limb ischemia, amputations.	Mixed
Wang et al., 2023 (12)	China	Surveillance analysis	278 IMD cases (all ages)	C (41%), B (32%), W (15%)	Varied (meningitis, meningococcemia)	Not detailed in summary.	Highlights rising serogroup B prevalence in the region of the present case.	Mixed
Baloche et al., 2022 (13)	France	Prospective cohort (protocol)	100 (0–15 yr)	Not specified	Long-term IMD sequelae evaluation	Standard care + follow-up	Ongoing study	Mixed
Inceli et al., 2025 (14)	Turkey	Case report	1 (6-month female)	B	Septic shock, PF, MODS	PICU support, antibiotics	Fatal	Severe only
Present Case, 2024	China	Case report	1 (12-year male)	B	PF, refractory shock, DIC, MODS (cardiac, renal)	Meropenem, vasopressors, ventilation, PLEX, CRRT	Survival with extensive limb scarring, skin grafting, persistent limp.	Severe only

Nm, Neisseria meningitidis; PF, purpura fulminans; DIC, disseminated intravascular coagulation; MODS, multiple organ dysfunction syndrome; PLEX, plasma exchange; CRRT, continuous renal replacement therapy.

This favorable tissue outcome is consistent with the hypothesis that early multimodal blood purification (TPE combined with CRRT) may attenuate microvascular thrombosis and modify sequelae distribution in purpura fulminans. However, given the uncontrolled nature of a single case report (*n* = 1), these observations cannot establish direct causality and should be regarded as hypothesis-generating.

### 3.7 Limitations

Several limitations of this study must be acknowledged:

Uncontrolled Single-Patient Design (*n* = 1): Causal inferences regarding the specific therapeutic efficacy of multimodal interventions (such as TPE and CRRT) in driving survival or limb preservation cannot be definitively established or generalized to broader clinical populations.

Absence of Isolate Genotyping: Neither whole-genome sequencing (WGS) nor Meningococcal Antigen Typing System (MATS) assays were performed on the recovered N. meningitidis isolate, preventing direct verification of antigenic matching or divergence between the patient's strain and available recombinant vaccines (e.g., 4CMenB).

Narrative Review Design: Although structured inclusion/exclusion criteria were applied, the comparative literature synthesis is narrative in scope rather than a formal systematic review or meta-analysis.

Unmeasured Immunological Status: Waning bactericidal antibody titers from historical polysaccharide vaccinations were not quantitatively measured at presentation, representing an unmeasured confounding factor when evaluating host susceptibility.

## Data Availability

The original contributions presented in the study are included in the article/Supplementary Material, further inquiries can be directed to the corresponding author.
